# Psychological consequences and associated risk factors among adult survivors of the 2008 Wenchuan earthquake

**DOI:** 10.1186/1471-244X-14-126

**Published:** 2014-04-29

**Authors:** Zhibin Wu, Jiuping Xu, Lili He

**Affiliations:** 1 Uncertainty Decision-making Laboratory, Sichuan University, 610064 Chengdu, China; 2 School of Business, Sichuan University, 610064, Chengdu, China

**Keywords:** Wenchuan earthquake, Post-traumatic stress disorder (PTSD), Anxiety, Depression, Risk factor

## Abstract

**Background:**

In 2008, a devastating earthquake measuring 8.0 on the Richter scale struck Wenchuan, China. Following this disaster, several studies were conducted which assessed the degree of mental disorders in the affected population, but very few considered that several disorders may occur at the same time. This paper aims to investigate the psychological effects and risk factors among adult survivors one-year after the earthquake event.

**Methods:**

2080 adult earthquake survivors from 19 counties in the affected areas were interviewed. A stratified sampling strategy was used to collect the information. Earthquake survivors completed self-report questionnaires, which included a post-traumatic stress disorder (PTSD) checklist, a self-rating depression scale and a self-rating anxiety scale.

**Results:**

Fifty nine percent of the participants were male. The prevalence of probable PTSD in the sample was 40.1% (based on the DSM-IV criteria). Significant differences in the demographic variables were found in the levels of anxiety, depression, and PTSD. Anxiety levels were found to be positively correlated with depression (r = 0.438, p < 0.01) and PTSD (r = 0.322, p < 0.01). Risk factors for each symptom were also identified. Being female, having a low income level and having a low perceived level of social support were found to be the risk factors associated with anxiety, depression, and PTSD. There appeared to be no obvious relationship between the distance from the epicenter of the earthquake event and the severity of the psychological problems.

**Conclusions:**

PTSD, anxiety, and depression were prevalent among the survivors. Most findings on the predictors were found to be consistent with current research. Positive adjustment and social support were found to be needed for the highest-risk population.

## Background

Earthquakes, which can cause widespread devastation, are life threatening, unpredictable and uncontrollable natural disasters. The catastrophic Wenchuan earthquake measuring 8.0 on the Richter scale occurred in the west of the Sichuan basin, China, and was China's second deadliest earthquake in recent history. Nearly 70,000 people were killed, 373,000 injured and 18,000 listed as missing, precipitating the largest crisis intervention in the history of China. The epicenter was 80 kilometers northwest of Chengdu, the capital of Sichuan province. In the aftermath of the earthquake, with the loss of loved ones, homelessness, and displacement, survivors had an increased risk of experiencing psychological issues.

The past two decades have seen an increase in research which documents the mental health effects of natural and man-made disasters [1-3]. Such disasters have been found to be associated with an increased prevalence in severe psychiatric symptomatology, stress, anxiety, depression, somatic complaints, and nightmares [4,5]. It has also been found that people’s behavior in the face of natural hazards can be deeply influenced by the cultural, social, economic, and political context [6]. These cultural variations have been used to examine how people react to significant and improbable outcomes in life [7]. However, it has also been documented that earthquake events in developed countries may cause less psychological distress among survivors than those in less developed countries because of the presence of more resources and, in particular, the availability of developed social support systems [8,9]. Further, longitudinal studies have shown that there is often a decline in symptomatology with the passage of time [10,11]. In cross-sectional research, many papers have focused on those factors which may be predictive for the development of psychological problems. Being female, having multiple stressors, displaying negative coping, and having a higher exposure to the disaster event have all been shown to be predictive of psychological problems [12-14]. The psychological consequences of earthquakes can be serious and persistent, even if the magnitude of the earthquake was moderate [15-17].

Exposure to earthquake events has been associated with psychological distress and in particular the development of post-traumatic stress disorders (PTSD). Of all the psychological problems resulting from natural disaster events, PTSD appears to be the most studied and reported consequence [10-13,18-20]. Galea and colleagues [21] have provided an excellent review of the empirical literature in this field. The results of the above studies have suggested that PTSD symptoms are common in earthquake survivors, with the prevalence of PTSD reported in victims of earthquake trauma ranging from 10% to 87% [10]. For example, 65% of participants were shown to have PTSD symptoms three years after the 2005 Pakistani earthquake [13] and after the Sichuan earthquake, a total of 62.8% of participants met the criteria for PTSD 1 month after the earthquake [20]. Kun and colleagues reported that the prevalence of suspected PTSD was 45.5% in the heavily damaged Beichuan County and 9.4% in the moderately damaged Langzhong County two and half months after the earthquake [18]. Wang and colleagues found that the probable prevalence of PTSD was 37.8% in the heavily affected communities and 13.0% in the moderately affected communities three months after the Sichuan earthquake [22]. The PTSD rate in college students in the severely affected area was 14.1% [23]. A study on adolescents showed that PTSD, anxiety, and depression were highly comorbid [24]. The variation in the results of these studies is because of the differences in the methodologies used, the sample population and the difference in the time post earthquake when the research was conducted.

In addition to PTSD, depression and anxiety have also been found to be common disorders in earthquake survivors. A study conducted after the Pakistani earthquake found that 63% of the earthquake-affected women of reproductive age had anxiety and 54% showed signs of depression [25]. Eight months after the Sichuan earthquake, the prevalence of depression was found to be 29.0% among postpartum women [26]. Goenjian and colleagues found that 32-months after the 1999 Parnitha earthquake in Greece, 13.6% of adolescents still met the criteria for clinical depression. Another study on the 1988 Armenian earthquake reported that 52% of adult survivors met the criteria for depression [11]. Corresponding risk factors were identified in these studies for the different groups being surveyed [26,27]. For example, unemployed women, those with a lower monthly family income, and those who suffered from poor sleep were found to have a higher risk of depression [26]. Knowledge of which segments of the population are most at risk can help agencies more effectively provide assistance in the aftermath of such events.

Up until now, the relationships between PTSD, depression and anxiety in Wenchuan earthquake survivors has not been well documented. This study aims to examine the prevalence and risk factors for PTSD, depression and anxiety in adult survivors in the aftermath of the Wenchuan earthquake event.

## Methods

### Participants

All participants in the study were urban residents who were living in either their original houses or in temporary accommodation. A high response rate was assured because of the assistance of the relevant government departments. A total of 2300 individuals were involved in the survey, with 2080 completing the questionnaire, a response rate of 90.4%. Participants were all selected from earthquake-stricken areas in two provinces — Sichuan and Shaanxi. In Sichuan Province, eighteen counties were selected: Dujiangyan, Pengzhou, Chongzhou, Shifang, Mianzhu, Jiangyou, Anxian, Pingwu, Beichuan, Jiange, Qingchuan, Hanyuan, Wenchuan, Lixian, Maoxian, Songpan, Heishui, and Xiaojin. In Shaanxi province, Lueyang was selected. The inclusion criteria were as follows: having a high degree of exposure to earthquake such as feeling the shock waves, and witnessing the aftermath. The study was designed in accordance with the tenets of the Declaration of Helsinki and was approved by the Sichuan University Ethics committee.

### Procedure

Data collection was conducted one year after the Wenchuan earthquake event, and a stratified random sampling strategy was adopted. In the first sampling stage, the 19 hardest hit counties were selected due to their high degree of exposure to the earthquake. In the second sampling stage, with the assistance of the local civil affairs department and Bureau of Statistics, several local communities were selected. In each community, households were randomly selected on the basis of the total number of the houses and temporary accommodation, and one adult within each selected house or temporary accommodation was randomly selected. From July to September, 2009, 19 trained groups, each made up of two postgraduate students and a staffer from the civil affairs department, were assigned to the nineteen counties based on a schedule arranged in advance. Each team has at least one member who was fluent in the local dialect. They visited households who were either living in their original houses or were in temporary accommodation. After a detailed explanation of the procedures, all participants gave their informed consent to participate in the survey. Group members explained the goals of the study to the subjects and made it clear that anonymity would be ensured. Very few participants were found to have a low education level or literacy problems, but for those who did, group members read the content of the questionnaire aloud and explained what each item meant, and, if necessary, also noted down the participant’s answers and assisted them throughout the survey process. There was less than 1% missing data in the collected data.

### Measures

The self-report questionnaire covered items on demographic characteristics, a self-rating depression scale, a self-rating anxiety scale, and a PTSD scale.

The demographic characteristics collected were: age, gender, ethnic group, education level, income level and housing type (Table 1).

**Table 1:** The characteristics of the study sample (N = 2,080)

	***N***	***%***		***N***	***%***
**Gender**			**Ethnic group**		
Male	1,227	59.0	Han	1,674	80.5
Female	853	41.0	Tibetan	147	7.1
			Qiang	211	10.1
**Age groups**			Other	48	2.3
18-30	441	21.2			
31-40	849	40.8	**Education level**		
41-50	609	29.3	No degree^a^	1095	52.6
51-68	181	8.7	Bachelor	938	45.1
			Graduate	47	2.3
**Income (RMB/month)**					
<1000 RMB	385	18.5	**Housing status**		
1000-2000 RMB	1360	65.4	Original house	758	36.4
2000-3000 RMB	264	12.7	Public dormitory	295	14.2
>3000 RMB	71	3.4	Rented house	643	30.9
			Temporary accommodation	384	18.5

^a^In China only undergraduates and above have a degree, so in our study all education lower than undergraduate level (including college diploma, high school and less than high school) are included in “No degree” group.

Social support was defined as “the assistance and protection given to others, especially to individuals” [28]. The Chinese version of the social support rating scale (SSRS) developed by Xiao [29] was used. This scale measures three dimensions of support: subjective support (4 items), objective support (3 items), and support availability (3 items). Subjective support focuses on the perceived interpersonal network that an individual can count on, objective support focuses on the degree of actual support an individual had received and support availability looked at an individual’s pattern of behavior when seeking social support. The SSRS has been previously applied to a wide range of Chinese populations because of its high reliability and validity [29,30]. The Cronbach alpha coefficient for the SSRS in this study was 0.91.

The self-rating depression scale (SDS) developed by Zung [31] is a 20-item self-report questionnaire that has been widely used as a screening tool, and covers the affective, psychological and somatic symptoms associated with depression. On the scale, there are ten positively worded and ten negatively worded questions, with each question being scored on a scale of 1 to 4 (1 = a little of the time, 2 = some of the time, 3 = a good part of the time, 4 = most of the time). According to Zung [31], the degree of depression can be measured by an index equal to the SDS score/80 (the total points) with ranges from 0.25 to 1.0. This index has 4 clinical categories: low depression (lower than 0.5); moderate depression (0.50–0.59); moderate to severe depression (0.60–0.69) and severe depression (higher than 0.70). The internal reliability of the SDS in this study using Cronbach’s alpha coefficient was 0.79.

The self-rating anxiety scale (SAS) which was also developed by Zung [32] is a simple test that determines the severity of the anxiety symptoms. The SAS has 20 questions which can be rated from 1 to 4 (1 = a little of the time, 2 = some of the time, 3 = a good part of the time, 4 = most of the time). Most of the questions are scaled, with the numbers increasing as symptoms worsen. Both the SDS and SAS were translated into Chinese and have been validated and widely used in China [29]. The degree of anxiety can be measured by an index equal to the SAS score/80 (the total points) with ranges from 0.25 to 1.0. The anxiety index also has 4 clinical categories: low anxiety (lower than 0.5); moderate anxiety (0.50–0.59); moderate to severe anxiety (0.60–0.69) and severe anxiety (higher than 0.70). The internal reliability of the SAS using Cronbach’s alpha coefficient was 0.83. The total scores for depression, anxiety and social support are used in the discussion in this paper.

PTSD symptoms were assessed using the PTSD Check List-Civilian Version (PCL-C) which has 17 items, with each one corresponding to a symptom in the Diagnostic and Statistical Manual of Mental Disorders, Fourth Edition (DSM-IV) PTSD criteria B, C, and D [33]. The PCL is one of the most widely used PTSD measures, and its psychometric properties have been well documented [23,34-36]. Each symptom was measured on a scale of 1 to 5 (rated 1 = not at all, 2 = slight, 3 = moderate, 4 = severe, 5 = extremely severe). Criteria B measured the re-experiencing level (5 symptoms), criteria C measured the levels of avoidance and emotional numbing (7 symptoms), and criteria D measured levels of increased arousal (5 symptoms). The self-report scores for each symptom in each criterion were totaled to measure the scale. The sum score derived from the ratings on the 17 individual symptoms allowed for a quantification of the PTSD severity. The internal reliability of this measure using Cronbach’s alpha coefficient was 0.88.

### Statistical analysis

Due to different purposes various statistical methods were utilized that are consistent with other research [13,23]. Data were expressed as a frequency for the nominal variables and as mean ± standard deviation (SD) for the continuous variables. One way ANOVA was used to examine differences among demographic variables. The post hoc tests were follow-ups to examine particular differences for the groups that had been determined to have significant differences. Multiple regression analysis was used to determine the predictors for the severity of psychological problems. A hierarchical clustering method was used to analyze regional differences. List wise deletion was used for missing data, and a p value 0.05 was used as the cut-off score. The statistical analyses were conducted using SPSS 16.0 software.

## Results

### Demographic characteristics

The mean age of the 2,080 participants at the time of the interview was 38.24 ± 8.82 years (ranging from 18–68 years), with the majority (i.e., 59%) being male and with over half (53%) having no degree. Apart from the Han ethnic group, the following ethnic groups participated in the survey: Tibetan, Qiang, and a small group which included the Hui, Tujia and Yi. Gender was coded 1 (male) and 2 (female). Age was divided into four groups: 18 to 30 (coded as 1), 31 to 40 (coded as 2), 41 to 50 (coded as 3), older than 51 (coded as 4). Ethnic groups were also listed and coded 1–4 which referred to the Han, Tibetan, Qiang, and Other respectively. The level of education was coded 1–3, that is, 1 = No degree, 2 = Bachelor, 3 = Graduate. Income was divided into four income levels, namely, 1 = less than 1000 Yuan, 2 = from 1000 to 2000 Yuan, 3 = from 2000 to 3000 Yuan, 4 = more than 3000 Yuan. In terms of housing, 1 = original house, 2 = public dormitory, 3 = rented house, 4 = temporary accommodation (Table 1).

### Basic data analyses

Scores on the SAS test were: 20–39 (41.6%), 40–47 (27.4%), 48–55 (20.5%), and 56–80 (10.5%). Scores on the SDS test were: 20–39 (46.1.0%), 40–47 (33.5%), 48–55 (13.4), and 56–80 (7.0%). For PTSD scores, 58.7% of participants reported 1 or more reexperience symptoms (an item with a score of 3 or more), 47.4% of participants reported 3 or more avoidance symptoms (an item with a score of 3 or more), and 40.1% of participants reported 2 or more arousal symptoms (an item with a score of 3 or more). The prevalence estimate of probable PTSD in our sample was 40.1% (based on the DSM-IV criteria).

By conducting one-way ANOVA, significant differences were found for the anxiety, depression, and PTSD on almost all the demographical variables (see Table 2).

**Table 2:** Test of mean difference scores (N = 2,080)

	***SAS***		***SDS***		***PTSD***	
	**Mean score**	***p*** **value**	**Mean score**	***p*** **value**	**Mean score**	***p*** **value**
**Gender**		<0.001***		0.048*		<0.001**
Male	40.62		40.50		39.64	
Female	44.40		41.25		41.62	
**Age**		0.083		0.381		0.039*
18-30	42.15		40.15		38.63	
31-40	42.93		41.02		39.93	
41-50	42.88		41.03		40.67	
51-68	44.07		40.90		41.05	
**Ethnic group**		0.018*		0.009**		0.047*
Han	43.71		41.14		40.58	
Tibetan	42.13		39.71		39.00	
Qiang	42.99		40.14		41.24	
Other	38.30		36.63		36.11	
**Education level**		0.012*		0.002**		0.005**
No degree	43.36		41.12		40.81	
Bachelor	43.69		40.58		40.31	
Graduate	38.76		36.89		35.94	
**Income (RMB/month)**		<0.001***		<0.001***		<0.001***
<1000 RMB	45.00		41.10		42.05	
1000-2000 RMB	44.05		41.32		40.80	
2000-3000 RMB	39.74		38.46		37.97	
>3000 RMB	37.15		37.41		34.58	
**Housing status**		<0.001***		<0.001***		<0.001***
Original house	42.46		40.41		39.47	
Public dormitory	42.92		39.45		40.24	
Rented house	45.22		42.85		42.42	
Temporary settlement	46.16		42.43		43.33	

*p <0.05. **p <0.01. ***p <0.01.

SAS: Self-rating Anxiety Scale; SDS: Self-rating Depression Scale; PTSD: Post-traumatic stress disorder.

In order to show which subcategory was associated with higher symptoms, the Tukey post-hoc comparisons were conducted. The results for post-hoc analysis are presented in Table 3. Tukey post-hoc tests revealed that the two lower income groups (income <1000 RMB and income 1000–2000 RMB) got significantly (p < 0.05) higher scores than the two higher groups (income 2000–3000 RMB and income >3000 RMB) in SAS, SDS and PTSD. With respect to education level, no significant differences were found between the “No degree” group and “Bachelor” group, but both groups got significantly (p < 0.05) higher scores than the “Graduate” group. It was found that for Housing status there were no significant differences between those who lived in their original house and those who lived in the public house, and there were also no significant differences between the “rented” group and “temporary” group. Note that the results of variables age and ethnic were omitted here. No significant post-hoc effects were found for age groups, although the difference between 31–40 group and 51–68 group in PTSD score were marginally significant. The Han ethnic group got significantly (p < 0.05) higher SAS and SDS scores than the “Other” ethnic group.

**Table 3:** The results of post-hoc analysis (N = 2,080)

		**SAS**		**SDS**		**PTSD**	
**I**	**J**	**Mean difference (I-J)**	***p***	**Mean difference (I-J)**	***p***	**Mean difference (I-J)**	***p***
**Education level**							
No degree	Bachelor	-0.33	0.787	0.54	0.371	0.49	0.560
	Graduate	4.61	0.015*	4.23	0.002**	4.86	0.004**
Bachelor	No degree	0.33	0.787	-0.54	0.371	-0.49	0.560
	Graduate	4.93	0.008**	3.69	0.008**	4.37	0.011*
Graduate	No degree	-4.61	0.015*	-4.23	0.002**	-4.86	0.004**
	Bachelor	-4.93	0.008**	-3.69	0.008**	-4.37	0.011*
**Income (RMB/month)**							
<1000	1000-2000	0.95	0.437	-0.21	0.976	1.25	0.180
	2000-3000	5.26	<0.001***	2.65	0.001**	4.08	<0.001***
	>3000	7.86	<0.001***	3.69	0.004**	7.47	<0.001***
1000-2000	<1000	-0.95	0.437	0.21	0.976	-1.25	0.180
	2000-3000	4.31	<0.001***	2.86	<0.001***	2.83	0.001**
	>3000	6.90	<0.001***	3.90	0.001**	6.22	<0.001***
2000-3000	<1000	-5.26	<0.001***	-2.65	0.001**	-4.08	<0.001***
	1000-2000	-4.31	<0.001***	-2.86	<0.001***	-2.83	0.001**
	>3000	2.59	0.250	1.04	0.787	3.39	0.086
>3000	<1000	-7.86	<0.001***	-3.69	0.004**	-7.47	<0.001***
	1000-2000	-6.90	<0.001***	-3.90	0.001***	-6.22	<0.001***
	2000-3000	-2.59	0.250	-1.04	0.787	-3.39	0.086
**Housing status**							
Original	Public	-0.45	0.891	0.95	0.224	-0.78	0.592
	Rented	-2.76	0.007**	-2.44	0.003**	-3.87	<0.001***
	Temporary	-3.69	<0.001***	-2.02	0.002**	-2.96	<0.001***
Public	Original	0.45	0.891	-0.95	0.224	0.78	0.592
	Rented	-2.31	0.072	-3.40	<0.001***	-0.39	0.003**
	Temporary	-3.24	<0.001***	-2.98	<0.001***	-2.18	0.031*
Rented	Original	2.76	0.007**	2.44	0.003**	3.87	<0.001***
	Public	2.31	0.072	3.40	<0.001***	3.09	0.003**
	Temporary	-0.93	0.781	0.42	0.954	0.91	0765
Temporary	Original	3.69	<0.001***	2.02	0.002**	2.96	<0.001***
	Public	3.24	<0.001***	2.98	<0.001***	2.18	0.031*
	Rented	0.93	0.781	-0.42	0.954	-0.91	0.765

*p <0.05. **p <0.01. ***p <0.01.

SAS: Self-rating Anxiety Scale; SDS: Self-rating Depression Scale; PTSD: Post-traumatic stress disorder.

Bivariate correlation analysis showed that the SAS score, the SDS score, the PTSD score and social support were related to each other (Table 4). The severity of anxiety symptoms was significantly positively correlated with the severity of depression symptoms (r = 0.438, p < 0.01) and PTSD (r = 0.322, p < 0.01), but negatively correlated with social support. The severity of depression symptoms was also significantly positively correlated with the severity of PTSD (r = 0.292, p < 0.01). High levels of social support were related to less severe symptoms for all three measures: anxiety, depression and PTSD.

**Table 4:** Correlation coefficients among the variables (N = 2,080)

	**SAS score**	**SDS score**	**PTSD score**	**SSRS score**
SAS score	1.000	0.438**	0.322**	-0.415**
SDS score	0.438**	1.000	0292**	-0.312**
PTSD score	0.322**	0.292**	1.000	-0.278**
SSRS score	-0.435**	-0.312**	-0.278**	1.000

**correlation is significant at the 0.01 level (2-tailed). SAS score: the severity degree for anxiety. SDS score: the severity degree for depression. PTSD score: the severity degree for PTSD. SSRS score: the level of social support.

### Regression analysis

To investigate the factors associated with psychological problems, we conducted multiple regression analyses. Since there are nominal or categorical variables, it is necessary that these variables are recoded as dummy variables. All the demographic variables and the three dimensions of social support were entered as independent variables. The results of these multiple regression analyses are in Table 5.

**Table 5:** The results of multiple regression analysis (N = 2,080)

**Variables**	***B***	**S.E.B**	**Beta**	***R***^***2***^
**Dependent variable: anxiety**				0.236
**Gender** (female)	1.014	0.499	0.053*	
**Age**				
18-30 (ref.)				
31-40	-2.242	1.068	-0.102*	
41-50	-0.501	0.981	-0.026	
51-68	-0.509	0.987	-0.024	
**Income**				
<1000 RMB (ref.)				
1000-2000 RMB	-4.085	0.729	-0.210***	
2000-3000 RMB	-8.926	1.033	-0.335***	
>3000RMB	-11.567	1.535	-0.244***	
**Housing status**				
Original house	-2.709	0.687	-0.145***	
Public dormitory	-2.234	0.797	-0.096**	
Rented house	-0.124	0.953	-0.004	
Temporary settlement (ref.)				
Subjective support	-0.527	0.073	-0.224***	
Objective support	-0.711	0.169	-0.157***	
Support availability	-1.163	0.323	-0.132***	
**Dependent variable: depression**				0.296
**Age**				
18-30 (ref.)				
31-40	-1.755	0.839	-0.103*	
41-50	-1.120	0.760	-0.078	
51-68	-0.748	0.767	-0.047	
**Income**				
<1000 RMB (ref.)				
1000-2000 RMB	-1.640	0.559	-0.111**	
2000-3000 RMB	-4.130	0.796	-0.202***	
>3000RMB	-5.579	1.184	-0.158***	
**Ethnic group**				
Han (ref.)				
Tibetan	-1.311	0.756	-0.045	
Qiang	-0.997	0.682	-0.038	
Other	-4.032	1.513	-0.070**	
**Housing status**				
Original house	-1.868	0.542	-0.131**	
Public dormitory	-2.692	0.624	-0.154***	
Rented house	0.808	0.756	0.034	
Temporary settlement (ref.)				
Subjective support	-0.954	0.066	-0.475***	
Objective support	0.289	0.132	0.084*	
Support availability	-0.952	0.245	-0.144***	
**Dependent variable: PTSD**				0.174
**Gender** (female)	1.196	0.496	0.066*	
**Income**				
<1000 RMB (ref.)				
1000-2000 RMB	-3.307	0.724	-0.176**	
2000-3000 RMB	-6.231	1.048	-0.236***	
>3000RMB	-10.069	1.618	-0.209***	
**Housing status**				
Original house	-2.068	0.682	-0.115**	
Public dormitory	-1.615	0.804	-0.071*	
Rented house	1.570	0.944	0.054	
Temporary settlement (ref.)				
Subjective support	-0.519	0.070	-0.240***	
Support availability	-1.053	0.315	-0.126*	

Note: only significant predictor variables are shown.

*B*: coefficient, *S.E.B*: standardized error of coefficient, *Beta*: standardized regression coefficient.

*p <0.05. **p <0.01. ***p <0.01.

Results showed that 23.6% of the variance in the anxiety score could be accounted for by the variables assessed in this study. Significant predictors of anxiety included female gender, being between ages 31 and 40, having an income of greater than 1000 RMB, living in original house and public dormitory, and social support. The depression severity assessed with the SDS was predicted by being between ages 31 and 40, having an income of greater than 1000 RMB, the “other” ethnic group, living in original house and public dormitory, and social support (R2 = 0.296). For the PTSD severity, the identified variables were female gender, having an income of greater than 1000 RMB, living in original house and public dormitory, subjective support and support availability, which together explained 17.4% of the variance. From Table 5, we can see that females, participants with a lower income level, participants living in temporary accommodation or rented house and those with lower social support were more likely to have psychological problems.

### Regional differences

We investigated the relationship between geographical location and anxiety, depression and PTSD symptoms. Of the 19 counties surveyed in our projects, the above three psychological aspects were selected for the classification. The corresponding mean scores of these three aspects for the 19 counties are given in Table 6, and the clustering results are also shown in Table 6. Note that those with lower mean scores had a lower level of symptoms for anxiety, depression and PTSD.

**Table 6:** Clustering data and results for the 19 counties

**County**	**Anxiety**	**Depression**	**PTSD**	**Group**
Chongzhou	47.08	45.58	43.83	1
Pingwu	47.26	43.80	43.05	1
Beichuan	46.06	43.10	43.74	1
Qingchuan	47.11	43.93	44.98	1
Wenchuan	49.38	43.27	46.20	1
Shifang	42.62	40.99	41.12	2
Mianzhu	43.12	41.55	40.09	2
Jiangyou	44.31	41.51	40.70	2
Anxian	43.64	40.90	41.66	2
Hanyuan	41.34	40.68	41.36	2
Lixian	41.90	40.32	41.16	2
Maoxian	42.91	40.74	39.46	2
Xiaojin	43.74	40.98	40.23	2
Heishui	43.90	41.34	39.41	2
Dujiangyan	40.10	39.72	37.95	3
Pengzhou	40.57	38.72	35.63	3
Jiange	37.66	36.98	35.26	3
Songpan	34.49	35.26	34.21	3
Lueyang	36.86	38.63	35.54	3

Note: the values in the columns Anxiety, Depression and PTSD are the mean scores.

The symptom levels of the three groups were better in the order from Group 1 to Group 3 with regard to each index. Wenchuan, Qingchuan and Beichuan located in the Longmenshan seismic belt were most severely affected by the earthquake event. These three counties were classified as group 1 where participants were found to have more severe symptoms of anxiety, depression and PTSD. According to the division of the affected areas by the central government, the extremely hard-hit areas were: Wenchuan, Beichuan, Mianzhu, Shifang, Qingchuan, Maoxian, Anxian, Dujiangyan, Pingwu, and Pengzhou. Except for Dujiangyan, the extremely hard-hit areas appear in the first two groups. From the geographical distribution of these counties, we found no obvious relationship between the distance from the epicenter and the severity of the psychological problems.

## Discussion

Natural disasters, such as earthquakes, cause traumatic stress and affect the lives of many people across the world. The 2008 Sichuan earthquake in China was a catastrophic disaster and had a tragic impact on many survivors. One year after the disaster, the affected populations had not fully recovered. Quite a few people in our sample reported psychiatric disorders such as PTSD, anxiety and depression.

This study showed that the female gender experienced greater levels of psychological problems for anxiety, depression and PTSD and gender was a risk factor for anxiety and PTD. A previous study of the Wenchuan earthquake also confirmed that being female was a risk factor for anxiety, depression and PTSD [36]. These results were also consistent with previous earthquake surveys which found that being female was a predictor of mental issues such as depression, traumatic stress responses and anxiety [9,37,38]. One of the explanations for this is that women tended to review or recall the disaster more often than men [11]. We also found that age group was a significant factor for the prediction of anxiety and depression but not PTSD. Elderly survivors tended to report a lower quality of life in terms of physical capacity, psychological well-being after the earthquake [39]. One study on the Wenchuan earthquake suggested that the elderly were more likely to have PTSD symptoms and general psychiatric morbidity compared with younger adult survivors (22.5% vs. 8.0%, p = 0.001) [40]. Other research, however, has reported that age was not significantly related to PTSD and psychological adjustment [10,18]. This study also found that participants with different education levels had significantly different scores for the presence of anxiety, depression and PTSD. However, in the multiple regression analyses, education level was not found to be a predictor for any of the three symptoms. This was not consistent with previous studies where a lower education had been found to be related to higher psychological problems [16,17,38,41].

Participants with a lower level of income showed a higher propensity for anxiety, depression and PTSD. This finding is in parallel with other research findings where lower socio-economic status was found to be related to a higher severity of psychological problems such as PTSD and depression among earthquake survivor [2,18,38,41]. A previous result suggested that persons of low socio-economic status reported higher levels of depressive symptoms than their counterparts [42]. Generally, research has shown that the poor are more likely to suffer psychological difficulties after a disaster compared to the better-off.

Our findings indicated that differences in living arrangements revealed significant differences in the anxiety, depression and PTSD scores. Participants living in temporary housing had higher scores than those living in their original houses. Many studies have showed an association between relocation and psychological morbidity [43] and those living in temporary housing have been found to be more likely to develop psychological problems [16,44]. Chen and colleagues [16] observed that those living in a prefabricated house tended to predict psychiatric morbidity. In a study done on the effect of types of temporary accommodation on people affected by a 1997 earthquake in Italy it was found that those earthquake victims who had been assigned to houses rather than container homes were more satisfied with and more attached to their temporary homes and reported greater psychological wellbeing [45]. Being relocated to a different area, and living in temporary housing below normal standards of living tended to indicate a higher level of distress [46].

Social support appears to buffer the negative impact of stressful events. In our sample, high levels of social support were found to be associated with less severe symptoms for anxiety, depression and PTSD. From the regression analysis, it was found that both subjective support and support availability appeared to be important factors in the prediction of the severity of anxiety, depression and PTSD. This result is in line with previous studies [2]. Higher perceived social support has been associated with higher positive emotions [13], and, those with stronger social support were more likely to have better quality of life [30]. Social support research often differentiates social embeddedness, received social support, and perceived social support. The ability of perceived social support to protect disaster victims’ mental health has been repeatedly demonstrated [2]. In another Wenchuan earthquake survey, subjects reported a high positive appraisal of the government’s rapid response to the earthquake [47]. Zang and colleagues [47] speculated that a positive view of the government could have an influence on the outcome of psychotherapies.

We expected that proximity to the epicenter during the earthquake would result in higher rates of post-earthquake psychological problems. However, the cluster analysis revealed no evidence of an obvious relationship between the distance to the epicenter and the severity of psychological symptoms. We also found differing studies in the literature concerning epicenter proximity and rate of property damage [38,41,48]. It should be noted that epicenter proximity and rate of property damage are not the only information that is needed to study the relationship between psychological symptoms and the impact of the earthquake [41]. There are many other variables that may explain the earthquake impact: emotional coping, self-worth, and social support, etc. [10]. In our study, a one-to-one aid policy was implemented in the post-earthquake reconstruction phase, which meant that an unaffected province in China gave aid and assistance to one affected county. Such a strategy may be considered a kind of social support. Another possible reason is the distinct and complex geographic environment in the affected areas. Some counties are located in plains areas, some in hilly areas, and others in plateau areas. The Chinese often believe that human beings are shaped by the land around them and such distinct geography may serve as an explanation of these irregular relationships.

This study explored the psychological effects and associated risk factors among survivors of the 2008 Wenchuan earthquake. There were significant psychological differences between the subgroups in the study sample. Being female, being older, having an income less than 1000 RMB, and living in temporary housing were the groups more likely to have psychological problems. From our findings and those in previous studies, there are some implications which may assist in lessening the effect of mental health issues on earthquake survivors. It may be helpful to develop a standard operating procedure for psychiatric services after an earthquake to assist the survivors suffering from psychiatric impairment [49]. There are potential well-established treatments such as exposure therapy, stress inoculation training, eye movement desensitization and reprocessing (EMDR) and trauma-focused cognitive-behavioral therapy (CBT) for PTSD [47,50-53]. These treatments have been shown to consistently reduce symptoms and improve growth and well-being. For example, Narrative Exposure Therapy (NET) could be a possibly effective tool in treating post-earthquake traumatic symptoms in adult Chinese earthquake survivors [47]. Highly trained interventionists who provide multi-session interventions might mitigate posttraumatic symptomatology following traumatic events [54]. Affected populations may also benefit from governmental and nongovernmental programs that provide social and economic support, as suggested by earlier studies [55].

There are several limitations to this study. First, the survey in our study did not cover the rural areas. Therefore, the sampling method may not accurately represent the full range of affected populations in the disaster-hit areas. Specifically, most young people in the rural areas had left their homes in order to support their families, and only children and older people tended to be at home. Second, the use of retrospective measures means some of the study data may be vulnerable to recall bias. A third and a major limitation in this study is that all participants in the study experienced the earthquake, and these results were not compared to any control groups from non-earthquake areas.

## Conclusions

The findings of this study add to a small body of work on the psychiatric consequences following an earthquake. PTSD, anxiety, and depression have been shown to be prevalent among the survivors. Therefore, from these results, it could be concluded that postdisaster mental health recovery programs that including early identification, ongoing monitoring, preventive and intervention programs, and sustained psychosocial support are needed for the highest-risk populations, namely, females, older people, people with lower incomes and those living in temporary housing.

## Competing interests

None of the authors have any conflicts of interest, financial or otherwise, relevant to the content of this manuscript.

## Authors’ contributions

ZW participated in designing the study, contributed to data analysis, and drafted the paper. JX designed the study, made substantial contributions to data interpretation. LH contributed to the data analysis and paper writing. All authors read and approved the final manuscript.

## Pre-publication history

The pre-publication history for this paper can be accessed here:

http://www.biomedcentral.com/1471-244X/14/126/prepub
